# Efficacy and causal mechanism of an online social media intervention to increase physical activity: Results of a randomized controlled trial

**DOI:** 10.1016/j.pmedr.2015.08.005

**Published:** 2015-08-13

**Authors:** Jingwen Zhang, Devon Brackbill, Sijia Yang, Damon Centola

**Affiliations:** University of Pennsylvania, Annenberg School for Communication, Philadelphia, PA, USA

**Keywords:** Exercise, Internet, Network, Social media, Randomized controlled trial

## Abstract

*Objective*: To identify what features of social media – promotional messaging or peer networks – can increase physical activity. *Method*: A 13-week social media-based exercise program was conducted at a large Northeastern university in Philadelphia, PA. In a randomized controlled trial, 217 graduate students from the University were randomized to three conditions: a control condition with a basic online program for enrolling in weekly exercise classes led by instructors of the University for 13 weeks, a media condition that supplemented the basic program with weekly online promotional media messages that encourage physical activity, and a social condition that replaced the media content with an online network of four to six anonymous peers composed of other participants of the program, in which each participant was able to see their peers' progress in enrolling in classes. The primary outcome was the number of enrollments in exercise classes, and the secondary outcomes were self-reported physical activities. Data were collected in 2014. *Results*: Participants enrolled in 5.5 classes on average. Compared with enrollment in the control condition (mean = 4.5), promotional messages moderately increased enrollment (mean = 5.7, p = 0.08), while anonymous social networks significantly increased enrollment (mean = 6.3, p = 0.02). By the end of the program, participants in the social condition reported exercising moderately for an additional 1.6 days each week compared with the baseline, which was significantly more than an additional 0.8 days in the control condition. *Conclusion*: Social influence from anonymous online peers was more successful than promotional messages for improving physical activity. *Clinical Trial Registration*: ClinicalTrials.gov: NCT02267369.

## Highlights

• We conduct a 13-week social media-based exercise program.
• We examine the causal effects of promotional messaging and social influence.
• Promotional messages moderately increase enrollment in exercise classes.
• Anonymous social networks significantly increase enrollment in exercise classes.
• Social influence is more successful for improving physical activity.

## Introduction

Sedentary lifestyle is an escalating epidemic (Dunstan et al., 2012, Hamilton et al., 2008, Thorp et al., 2011). Despite the known health benefits of physical activity (Haskell et al., 2007, Warburton et al., 2006, Young et al., 2014a), less than 30% of American adults 18 to 24 years of age met the federal Physical Activity Guidelines in 2012 (National Center for Health Statistics, 2014). Due to major life event changes associated with the independence and work schedule, a high proportion of young adults are sedentary or irregularly active (Gordon-Larsen et al., 2004, Woods et al., 2002). This statistic is alarming because sedentary habits developed in younger ages are likely to continue into later life (Kvaavik et al., 2003).

Improved technologies for both broadcasting health messages (Chou et al., 2013, Kreps and Neuhauser, 2010) and providing supportive social influences (Centola, 2013) make social media a promising intervention platform for increasing physical activity. Although health providers and entrepreneurs have attempted to use social media for promoting health and fitness (Bennett and Glasgow, 2009, Chou et al., 2013, Korda and Itani, 2013, Li et al., 2013, Young et al., 2014b), recent meta-analyses have provided mixed support for its effectiveness (Laranjo et al., 2015, Maher et al., 2014). Little is known about whether or how social media can be used to design a cost-effective solution for sedentary lifestyles.

This paper addresses this problem and presents results from a randomized controlled trial (RCT) that evaluated two prominent strategies for conducting exercise interventions using elements of social media: motivational campaigns that use professionally produced messages to improve exercise habits (Kroeze et al., 2006, Mozaffarian et al., 2012, Williams and French, 2011), and peer networks that provide information about the behavior of other members in the online program (Centola, 2010, Centola, 2011).

Promotional messages have been argued to be effective intervention strategies (Bennett and Glasgow, 2009, Cassell et al., 1998). In particular, multimedia health campaigns that combine visual and audio components in a high arousal format have been effective for increasing responsiveness (Houts et al., 2006, Kang et al., 2006). Less is known, however, about how online social networks might impact physical activity. Effects of social influence on health behaviors and outcomes have been documented (Christakis and Fowler, 2013). Recent studies suggest that online peer networks may be effective for increasing fitness among social media users through elevating social influence (Centola, 2013). However, it is difficult to evaluate the causal effects of social networks on physical activity in previous RCTs because they typically combined multiple strategies such as health education, peer interaction, and motivational messaging into a single treatment, making it impossible to identify a specific mechanism directly associated with improved activity (Cavallo et al., 2012, Napolitano et al., 2013, Neiger et al., 2012). As the findings from this study show, identifying these mechanisms is an important first step for establishing a baseline model for effective online interventions.

## Methods

### Study design

A 13-week social media-based exercise program called SHAPE-UP was conducted at a Northeastern university. The program offered 49 workout classes to assist participants in establishing exercise routines. On average, four classes were offered per week at the University's gym. Most classes were offered every week, such as yoga, muscle building, and group exercising. Some classes were offered occasionally, such as running, Pilates, spinning, and weight lifting, depending on the schedule of the Department of Recreation and Health Services (DRHS) at the University. Each class lasted for an hour and was typically scheduled in the evening. The size of the classes varied depending on the class enrollment. On average, 20 students were in each class. Classes were led by instructors from DRHS, who were informed of the project but not included as a part of the strategy to increase program participation. Participation in all classes was restricted to the program participants.

The SHAPE-UP website was designed for participants to enroll in classes and to interact with the program. To use the website, each participant created an anonymous online profile. All participants had continuous and equal access to the website. Classes were pre-programmed into an online calendar. Upon clicking a class, participants could read a description and register for it. The registration then triggered a confirmation email that was immediately sent to the participant and a reminder email 12 h before the class started. In addition, an online tracking tool allowed participants to keep a daily journal of their class completion.

**Fig. 1:**
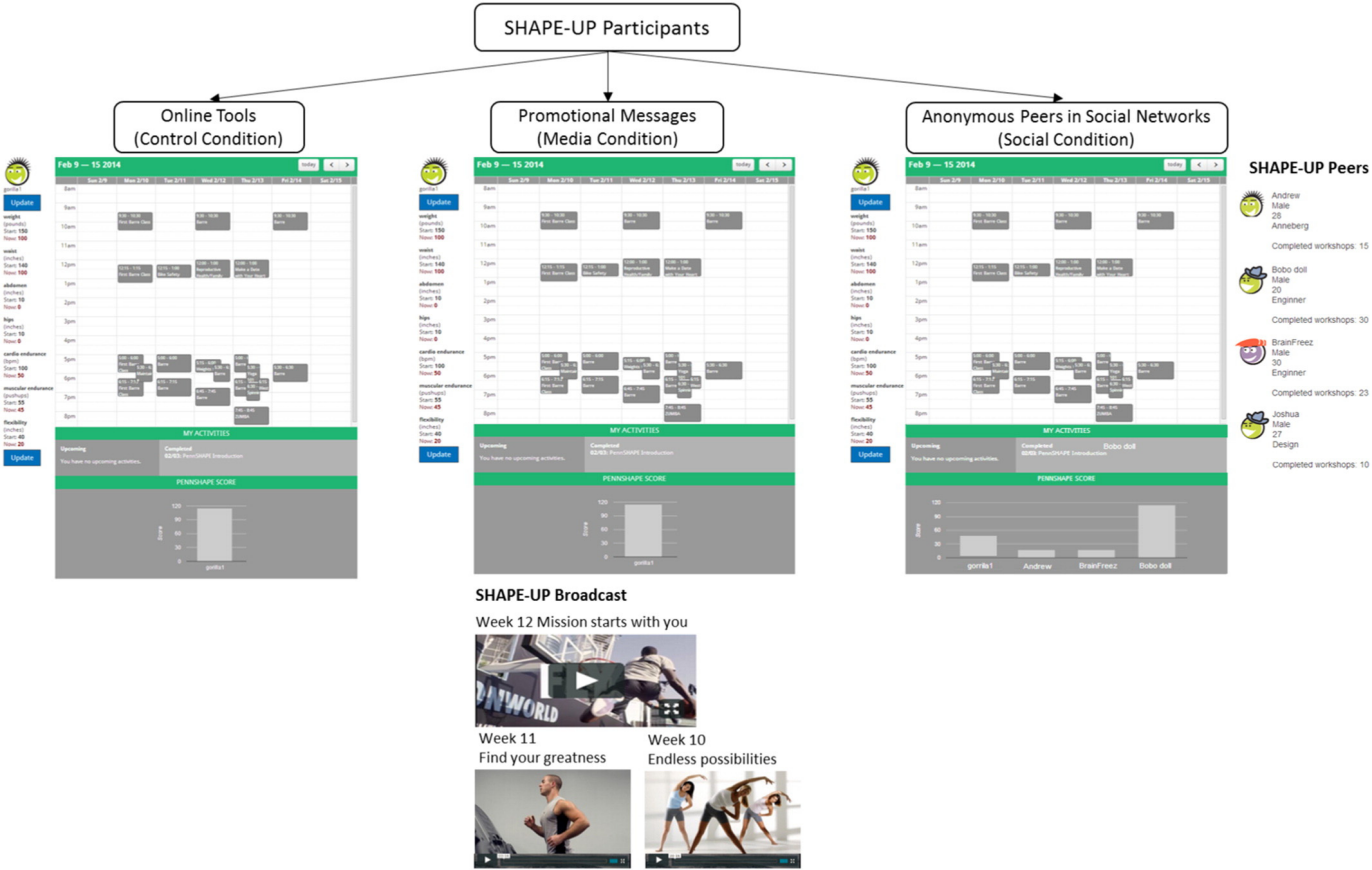
Example webpage illustrations for the three experimental conditions in the trial, Philadelphia, PA, 2014.

Upon using the website for the first time, participants were randomly assigned to one of three conditions: (1) The basic website (i.e., the control condition); (2) The basic website plus promotional messages (i.e., the media condition); and (3) The basic website plus social network (i.e., the social condition). Fig. 1 illustrates the three conditions of the experimental design.

The control condition provided participants with online tools for enrolling in classes and recording their progress. The media condition supplemented the basic tools with promotional media messages, including: two high arousal videos encouraging physical activity and one infographic with exercise tips and motivational messages on a weekly basis. The social condition, by contrast, omitted this media content. Instead, the basic website was supplemented with a network of four to six anonymous “peers”, composed of other participants of the program. Each participant was able to see their peers' profile information including username, gender, age, school, as well as information about their peers' progress, and real-time notifications about their peers' completion of classes. These networks did not provide any additional incentives or content to promote physical activity, nor could participants directly communicate with their peers through the website. This experimental design identifies the independent effects of promotional messaging and peer networks on increasing physical activity.

### Study participants

Participants were recruited through advertisements on the University's website, the student email list, and the Facebook page of a graduate student organization. In addition, flyers were put up on billboards in campus buildings. Recruitment materials specified that the purpose of the project was to improve the quality of student life by encouraging physical activity. Graduate students who were older than 18 years of age and who logged into the program website at least once after creating online profiles were eligible. In addition, eligibility was determined by an initial physical assessment conducted by the DRHS. Each participant first completed a screening questionnaire (Canadian Society for Exercise Physiology, CSEP, 2002) designed to identify adults for whom physical activity might be inappropriate, then completed a pushup test, a sit-and-reach test, and a 3-minute step test according to the YMCA fitness test (Franks and YMCA of the USA., 1989). No participant failed the assessment. All enrolled graduate students at the University were eligible to participate. The assessment lasted for 10 min and measured participants' heights and weights for calculating the Body Mass Index (BMI) (National Heart Lung and Blood Institute and National Institute of Diabetes and Digestive and Kidney Diseases, U.S., 1998).

Computer-generated random numbers were used to randomly assign participants to conditions. Participant enrollment and initial assessments were conducted from January 15, 2014 through February 1, 2014. Eligible participants completed a baseline online survey assessing demographic information and self-reported physical activity. Class instructors were blind to group assignments. All participants logged into the website throughout the 13 weeks at least once. At the end of the program, one week after all the classes were finished, they were contacted again for a post-program online survey. Data collection was completed by May 5, 2014. The study was approved by the institutional review board of the University, and all participants provided informed consent.

### Outcome measures

The primary outcome was the number of enrollments in exercise classes, which was recorded when participants digitally confirmed class registration. Class instructors confirmed the attendance of enrolled participants. The secondary outcomes were the changes from baseline to post-program in participants' self-reported number of days for moderate-cardiovascular (i.e., participating in physical activity for at least 30 min that did not make you sweat or breathe hard), intensive-cardiovascular (i.e., exercising or participating in physical activity for at least 20 min that made you sweat and breathe hard), and strength-building activities (i.e., doing exercises to strengthen or tone your muscles) in the previous 7 days assessed using the 3 questions developed by the Centers for Disease Control and Prevention (2001). Participants provided responses for the 3 questions at baseline and again one week after classes finished at the end of the program.

### Statistical analysis

A power analysis was performed to calculate the sample size required to detect a significant effect of the two treatment conditions on the primary outcome, class enrollment. Assuming a two-tailed test, α = 0.05, 20% attrition, and an effect size of Cohen's d = 0.6 (Cavallo et al., 2012, Foster et al., 2010, Valle et al., 2013), 55 participants were needed in each condition (44 after attrition) to ensure 80% power to detect a significant difference between treatment and control.

The means and distributions of class enrollment numbers were calculated in each condition, and confidence intervals were created from 10,000 bootstrap simulations. Enrollment numbers departed significantly from a normal distribution by a Shapiro–Wilk test (Shapiro and Wilk, 1965). Consequently, the non-parametric Wilcoxon rank sum test (two-tailed) (Efron, 1981, Gibbons, 1993) was used to examine the effects of the treatment conditions on overall enrollment compared with the control condition. Further analyses used logistic regression models to estimate the probability of enrollment in specific numbers of classes in the two treatment conditions compared with the control condition. Additionally, a linear regression model was constructed to examine changes in the rates of enrollment in the two treatment conditions compared with the control condition throughout the program.

Considering the secondary outcomes, the means and distributions of changes in self-reported numbers of days for moderate, intensive, and strength-building activities in the previous 7 days were calculated in each condition. Similar to the primary outcome, the secondary outcomes also departed significantly from normal distributions. Thus, the Wilcoxon rank sum tests (two-tailed) were used to examine the effects of the treatment conditions on the changes of three types of self-reported physical activities compared with the control condition.

All analyses were performed using an intent-to-treat mode applying the Last Observation Carried Forward (LOCF) (Shao and Zhong, 2003) method with participants analyzed based on their intervention assignment, regardless of the number of classes attended or completion of the follow-up assessment. All statistical analyses were conducted in R, version 3.1.0, in May 2014 through August 2014.

## Results

**Fig. 2:**
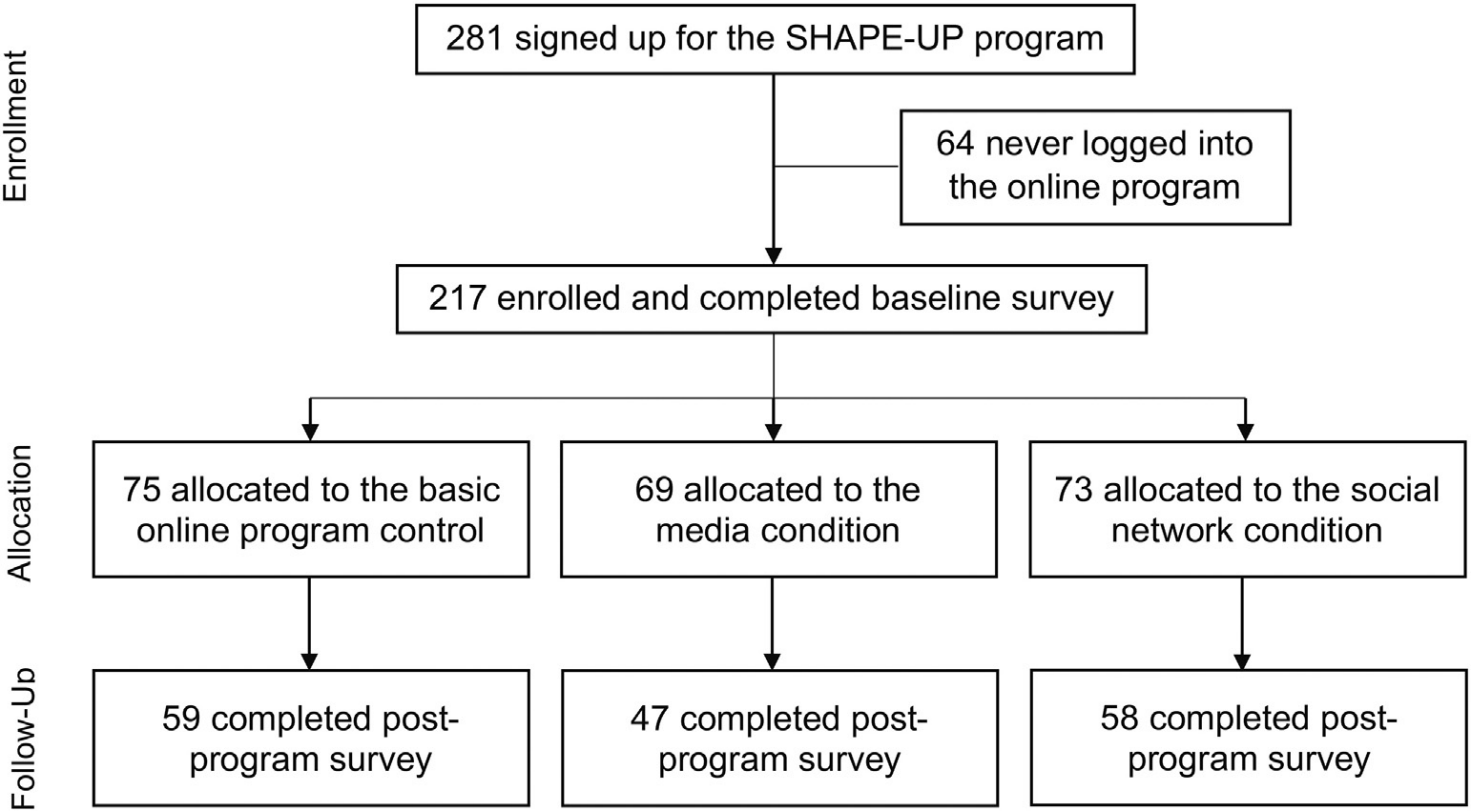
Flow diagram of participants through the trial, Philadelphia, PA, 2014.

A total of 281 graduate students signed up for the program and 217 were enrolled beginning in January 2014 and ending in May 2014. Fig. 2 shows the flow of participants from enrollment through the follow-up. In total, 164 (75.6%) completed the post-program survey. The attrition rate was not statistically different across conditions.

Table 1 shows participants' characteristics. There were no baseline gender, age, BMI, or physical activity level differences between participants across conditions. Participants ranged in age from 21 to 51 years (mean = 25.8, SD = 4.0) and ranged in BMI from 16.4 to 38.8 (mean = 23.9, SD = 4.4).

### Effects on exercise class enrollment

**Fig. 3:**
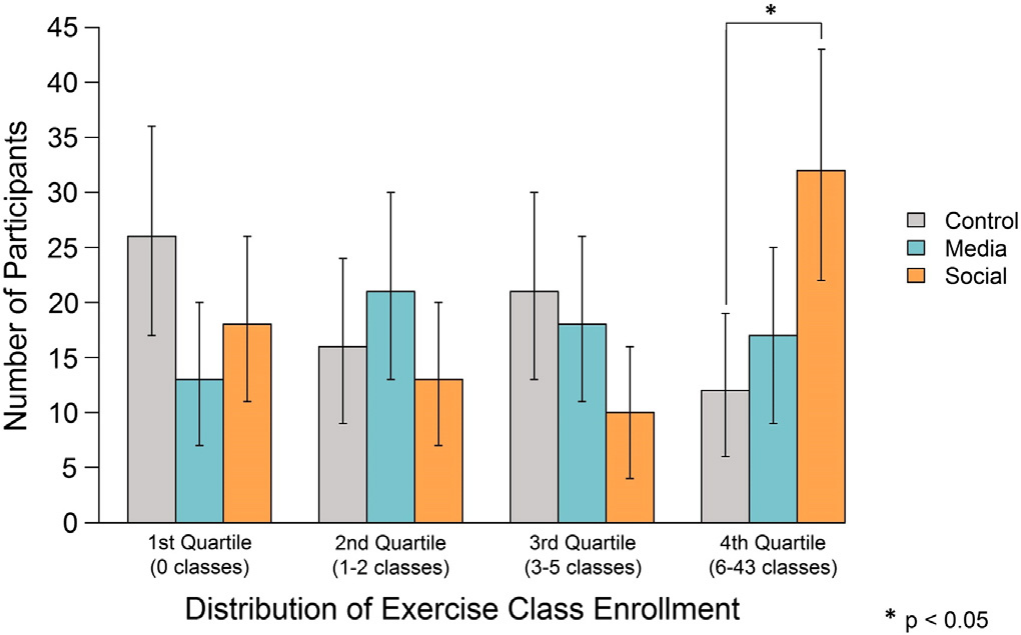
Distribution of exercise class enrollment across all quartiles by experimental condition, Philadelphia, PA, 2014.

Data from all 217 participants were used for analyses on the primary outcome. The overall mean number of enrollments was 5.5 (SE = 0.5) throughout the program. The highest level of enrollment occurred in the social condition (mean = 6.3, SE = 0.9), followed by the media condition (mean = 5.7, SE = 1.1), and the control (mean = 4.5, SE = 0.9). Enrollment numbers departed significantly from a normal distribution (Shapiro–Wilk W = 0.69, p < 0.001). Fig. 3 shows the distribution of enrollment numbers across all quartiles for each experimental condition.

The control condition had the largest fraction of participants in the first quartile (i.e., no enrollment). The media condition modestly shifted the distribution of enrollment away from the lowest quartile, increasing the fraction of participants in the upper enrollment quartiles by 18% as compared with the control condition (W = 5431, p = 0.08). However, overall class enrollment in the media condition was not significantly greater than the control condition.

By contrast, in comparison with the control condition, social influence significantly increased overall enrollment (W = 6048, p = 0.02, r = 0.20), producing a 167% increase in the fraction of participants above the 75th percentile of enrollment compared with the control condition (95% CI: 42% to 483%). There was no significant difference between the two treatment conditions.

**Fig. 4:**
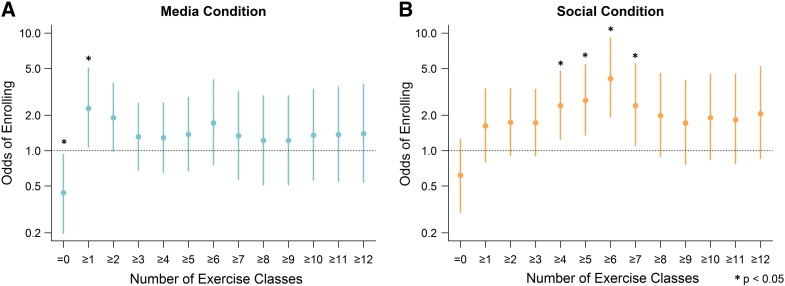
Effects of the media condition and the social condition on the likelihood of enrollment in increasing numbers of exercise classes, Philadelphia, PA, 2014.

A more detailed analysis shows that the effects of promotional messages and social influence differed in both strength and kind. Fig. 4 shows the odds ratios of participants enrolling in increasing number of classes in comparison with the control condition, beginning with the odds of enrolling in exactly zero classes (= 0), followed by the odds of enrolling in at least one class (≥ 1), at least two classes (≥ 2), and so on.

Panel A shows that promotional messages significantly increased the odds of enrollment among the least active participants. Participants in the media condition were significantly less likely to enroll in exactly zero classes than participants in the control condition, and were significantly more likely to enroll in at least one class. Promotional media thus was effective at increasing participation at the very low end, motivating non-participators to enroll in at least a single class. The effect size was small and attenuated after a single enrollment.

Panel B shows that social influence significantly increased participants' likelihood of enrolling in 4 or more, 5 or more, 6 or more, and 7 or more classes, as compared with the control condition. The social effect was stronger for greater levels of enrollment — increasing in magnitude from 4 or more classes up to 6 or more classes. As compared with the control condition, participants in the social condition were 60% more likely to enroll in 4 or more classes (OR = 2.4, p < 0.01), 80% more likely to enroll in 5 or more classes (OR = 2.7, p < 0.001), and 170% more likely to enroll in 6 or more classes (OR = 4.1, p < 0.001). The results suggest that promotional messages encouraged non-active people to try enrolling once. However, continued messaging did not have an enduring effect beyond that. Social influence, by contrast, significantly increased the likelihood of repeated enrollment.

**Fig. 5:**
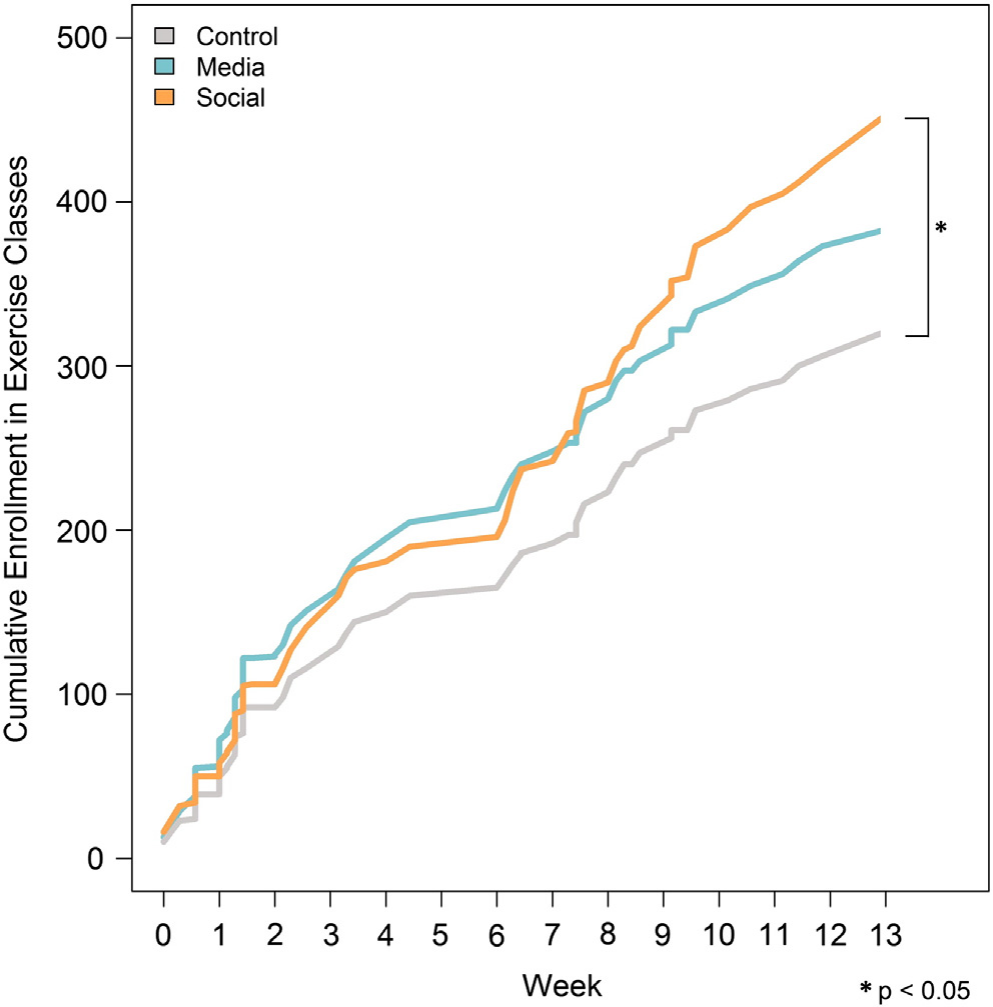
Cumulative enrollment in exercise classes by experimental condition, Philadelphia, PA, 2014.

In addition, the cumulative levels of enrollment in each experimental condition over the 13-week program were compared, as shown in Fig. 5.

In the first half of the program (i.e., through week 6), enrollment rates were significantly greater in both the media (6.42 per day, p = 0.001) and social conditions (6.15 per day, p = 0.01), than in the control condition (5.16 per day). The social and media conditions showed no significant difference from one another (p = 0.48). However, during the second half, average enrollment rates in the media condition slowed considerably (3.55 per day), showing no significant difference from the control (3.26 per day) (p = 0.24). By contrast, average enrollment rates in the social condition remained elevated (5.22 per day) and were significantly greater than both the control (p < 0.001) and media condition (p < 0.001). While the effects of promotional messages and social influence were comparable at the beginning of the program, social influence was significantly more effective at creating sustained engagement.

### Effects on daily exercises

By the end of the program, participants reported exercising for an additional 1.1 days (SE = 0.2) for moderate activities, 1.2 days (SE = 0.1) for intensive activities, and 0.5 days (SE = 0.1) for strength-building activities on average in the past week. These outcomes departed significantly from normal distributions. There was only one significant effect of the social condition on moderate activity compared with the control condition. Participants in the social condition reported exercising moderately for an additional 1.6 days (SE = 0.3) on average each week than at the baseline, compared with an extra 0.8 days (SE = 0.3) per week in the control (W = 2129.5, p = 0.02, r = 0.21). By contrast, participants in the media condition were exercising on average 0.9 days (SE = 0.3) more each week than at baseline, which was not significantly different from the control (W = 1437, p = 0.74). No significant effect was found on the intensive or strength-building activities. As a robustness test, we conducted the same analyses using data only from the 164 participants who completed the post-program survey and found consistent results.

## Discussion

This study reports findings on the efficacy of two key components of social media interventions in an exercise program. The findings highlight the different roles played by promotional messaging and social influence for encouraging behavior change. Promotional messages were effective for increasing initial engagement in exercise classes. However, these effects attenuated over time (Hornik, 2002, Randolph and Viswanath, 2004). By contrast, peer networks significantly improved participation levels while also increasing self-reported levels of engagement with physical activities. Taking these results together, they suggest that an effective strategy for using social media to promote behavior change may be to employ a hybrid approach that initially encourages physical activity through media campaigns, which are then replaced by program-generated peer influence networks that can encourage continued engagement.

In this study, real-time signals about peers' exercise behaviors constituted the main form of social influence, which may have helped to form participants' normative perceptions about the SHAPE-UP community (Cialdini, 2007, Cialdini and Goldstein, 2004). Our results suggest that peers knowing one another's levels of class enrollment may have helped them develop beliefs about the entire community's levels of activity and engagement in regular exercise. The strength of these social effects is particularly striking given the conservative implementation of the peer network. In contrast to previous work that used rich social environments such as Facebook, interactive chatting, and social support (Maher et al., 2014), the social networks used in this study were relatively minimal, as they were composed of anonymous, system-assigned online peers, without any capacity for direct communication.

Our methodological approach demonstrates an important innovation over traditional web-based studies that rely on using existing online applications to study large online populations (Dunton and Robertson, 2008, Lefebvre and Bornkessel, 2013). While traditional approaches cannot provide a clear identification of the effects of online networks on behavior change (Centola, 2013), our approach allowed us to construct peer-to-peer health networks in which we controlled all of the informational and social signals within the community. Our findings suggest that these methods may be feasibly used to deploy behavioral interventions through social media tools that are tailored to researcher-specific questions. Future research can extend this approach to construct online networks that causally identify the role of peer influence in decisions about preventative health screenings, vaccination, and medication compliance, thus leveraging the dynamics of social influence for understanding how networks affect both adoption and adherence to long-term health behaviors.

While our experimental design offered several methodological advantages, including the ability to create controlled longitudinal programming in an institutional setting and the capacity to identify the causal mechanisms through which social media directly impact health behaviors, these scientific advantages also come with limitations. Most notably, our program focused on graduate students, who are typically less at risk than other segments of the broader population. In future work, the experimental design could be extended to study other behaviors that may be more broadly applicable outside a university facility, such as running, walking or complying with prescribed medications. A second limitation is that our secondary outcomes were self-reported measures, which may be subject to inaccurate recall and social desirability bias. While these data provided a useful complement to the primary behavioral results, future studies would benefit from using behavioral measures of exercise intensity and time spent in exercises to supplement these self-reported measures. Finally, while our study focused on the effects of social media over a single semester, we did not conduct process evaluations on participants' engagement with either the promotional media, or the website interface for the study. Post-study interviews confirmed that participants in the media condition responded positively to exposure to promotional media. However, additional studies are required to provide deeper insight into the direct effects of promotional media signals on participants' subjective evaluations of their behavior. Future work would also benefit from extending our approach to include long-term follow-up data on participants' subsequent engagement with program-related exercises. While these limitations provide useful directions for future work, the approach developed in this study offers an important step forward for using in vivo experimental designs for identifying the effects of social media on health behaviors.

## Disclosures

None.

## Conflict of interest statement

The authors declare that there are no conflicts of interests.

## Figures and Tables

**Table 1:** Baseline demographic characteristics of participates by experimental condition, Philadelphia, PA, 2014.

	Control	Media	Social	Total
Participants (N)	75	69	73	217
Age (years; M [SD])	26.8 (4.8)	25.4 (3.6)	25.1 (3.1)	25.8 (4.0)
Male sex (%)	30.7	24.6	31.5	29.0
Body Mass Index (kg/m^2^; M [SD])	23.5 (4.1)	23.8 (4.4)	24.5 (4.7)	23.9 (4.4)
Overweight (BMI [25.0–29.9]; N [%])	19 (25.3)	17 (24.6)	13 (17.8)	49 (22.6)
Obese (BMI ≥ 30; N [%])	5 (6.7)	6 (8.7)	12 (16.4)	23 (10.6)
Met physical activity guideline in the past 7 days (N [%])	11 (14.7)	11 (15.9)	15 (20.6)	37 (17.1)
Moderate exercise in the past 7 days (days; M [SD])	2.2 (2.1)	2.0 (2.2)	1.8 (2.2)	2.0 (2.2)
Intensive exercise in the past 7 days (days; M [SD])	1.7 (1.6)	1.9 (2.0)	1.8 (1.8)	1.8 (1.8)
Strength exercise in the past 7 days (days; M [SD])	1.3 (1.5)	1.2 (1.6)	1.3 (1.7)	1.3 (1.6)

Note: no significant differences on all variables at baseline across conditions.
